# Design of highly active Ni catalysts supported on carbon nanofibers for the hydrolytic hydrogenation of cellobiose

**DOI:** 10.3389/fchem.2022.976281

**Published:** 2022-08-24

**Authors:** Esther Frecha, Javier Remón, Daniel Torres, Isabel Suelves, José Luis Pinilla

**Affiliations:** Instituto de Carboquímica, CSIC, Zaragoza, Spain

**Keywords:** cellobiose, hydrolytic hydrogenation, carbon supported catalyst, nickel, glucose, sorbitol, one pot reaction

## Abstract

The direct transformation of cellulose into sugar alcohols (*one-pot* conversion) over supported nickel catalysts represents an attractive chemical route for biomass valorization, allowing the use of subcritical water in the hydrolysis step. The effectiveness of this process is substantially conditioned by the hydrogenation ability of the catalyst, determined by design parameters such as the active phase loading and particle size. Herein, mechanistic insights into catalyst design to produce superior activity were outlined using the hydrolytic hydrogenation of cellobiose as a model reaction. Variations in the impregnation technique (precipitation in basic media, incipient wetness impregnation, and the use of colloidal-deposition approaches) endowed carbon-nanofiber-supported catalysts within a wide range of Ni crystal sizes (5.8–20.4 nm) and loadings (5–14 wt%). The link between the properties of these catalysts and their reactivity has been established using characterization techniques such as X-ray diffraction, transmission electron microscopy, X-ray photoelectron spectroscopy, and inductively coupled plasma-optical emission spectroscopy (ICP-OES). A fair compromise was found between the Ni surface area (3.89 m^2^/g) and its resistance against oxidation for intermediate crystallite sizes (∼11.3 nm) loaded at 10.7 wt%, affording the hydrogenation of 81.2% cellobiose to sorbitol after 3 h reaction at 190°C and 4.0 MPa H_2_ (measured at room temperature). The facile oxidation of smaller Ni particle sizes impeded the use of highly dispersed catalysts to reduce the metal content requirements.

## 1 Introduction

**SCHEME 1:**
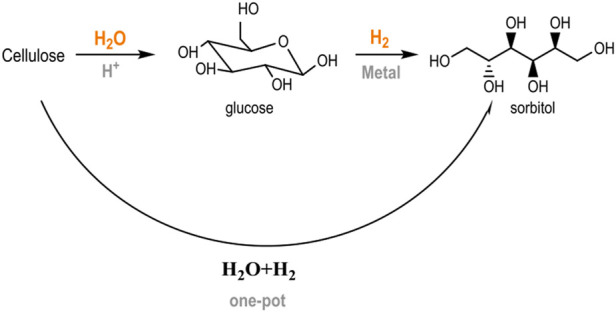
Catalytic conversion of cellulose into hexitols (adapted from Ribeiro et al. (2015)).

A current trend in second-generation biorefinery schemes is the operation of one-pot conversion systems, in which several catalytic reactions occur consecutively in a single stage (Vennestrøm et al., 2010; Climent et al., 2011; Climent et al., 2014). The advantages of using multi-step pathways include use of the synergies established between different chemical routes (tandem reactions) and using more compact manufacturing facilities without the necessity of intermediate separation steps (Delgado Arcaño et al., 2020). The hydrolytic hydrogenation of cellulose into sugar alcohols is an example of this transformation. This reaction combines an initial hydrolysis step with the simultaneous hydrogenation of glucose units into their metastable counterpart (sorbitol) in the presence of metal centers and a reductive atmosphere (Scheme 1) (Ribeiro et al., 2015). This approach circumvents the common acid/thermal sugar degradation that occurs under typical hydrolysis conditions, allowing it to function at relatively high temperatures (180–250°C) with high product selectivity (Dhepe and Fukuoka, 2008; Cabiac et al., 2011).

The final product is a versatile platform molecule that is listed among the top 10 valuable bio-based chemicals by the U.S. Department of Energy (DoE) (Werpy et al., 2004). It can be directly applied in the cosmetic and pharmaceutical industries as a sweetener and emulsifier (Kobayashi and Fukuoka, 2013). Further routes of upgrading enable its valorization to sorbitan and isosorbide through dehydration reactions or other polyols *via* hydrogenolysis. Isosorbide is used to synthesize fuel additives, surfactants, and bio-based plastics, where it is regarded as a surrogate compound of bisphenol A (Rose and Palkovits, 2012; Luterbacher et al., 2014; Liguori et al., 2020). Meanwhile, short-chain polyols are downstream products that have promising prospects in the petrochemical industry (Zhang et al., 2013; Liu et al., 2014).

The first studies of the production of polyols from polysaccharides date back to an initial study by Balandin et al. (1959) in the late 1950s, which reported the combination of mineral acids (H_2_SO_4_, H_3_PO_4_, and HCl) with a supported metal catalyst (Ru/C) (Vasyunina et al., 1960). In 2006, Fukuoka and Dhepe pioneered the conversion of cellulose into sugar alcohols in the aqueous phase over a bifunctional catalyst (Pt/γAl_2_O_3_) with a yield to hexitols of 31% under optimized conditions (24 h at 190°C and 5.0 MPa H_2_). In that system, Pt promoted hydrogenation and hydrolysis through the generation of protic sites by splitting H_2_ onto the metal surface and allowing subsequent spill over to the support (Fukuoka and Dhepe, 2006; Jollet et al., 2009). The intrinsic acidity of the support (γ-Al_2_O_3_) likely contributed to the hydrolysis of cellulose, although its hydration into boehmite (AlO(OH)) prevented catalyst recycling (Kobayashi, Ito, Komanoya, Hosaka, Dhepe, Kasai, Hara, and Fukuoka). Similarly, Luo et al. (2007) applied elevated temperatures to reversibly generate *in situ* H^+^ ions from hot water dissociation in the presence of ruthenium nanoclusters supported on active carbon (Ru/C) for hydrogenation. After 30 min at 245°C and 6.0 MPa H_2_, a 39% yield of C6 sugar alcohols was attained at 85% conversion. The hydrolysis activity was entirely ascribed to acid sites created under hydrothermal conditions as almost equal cellulose conversion (87.5%) was measured in non-catalyzed control experiments. More convincingly, no increase in initial cellulose conversion was noted, irrespective of the metal loading (38.1% and 37.9% conversion after 5 min of reaction for 1 and 8 wt% Ru/C, respectively).

Since that time, extensive research has been devoted to enhancing process selectivity toward target polyols, through improved catalyst design and/or by carefully selecting optimal reaction conditions (Matthiesen et al., 2014). An assortment of materials have been tested as catalytic supports, including acid zeolites (Geboers et al., 2011; Negoi et al., 2014), metal oxides such as silica (Reyes-Luyanda et al., 2012), alumina (Kobayashi et al., 2011), and ceria-based mixed oxides (Dar et al., 2015) or carbon materials (Wang et al., 2012a; Han and Lee, 2012; Park et al., 2013; Zhao et al., 2015). Generally, the use of graphitic materials has several catalytic advantages over other options, such as hydrothermal stability, mechanical strength, large surface area, and tunable surface chemistry (Navalon et al., 2016; Hu et al., 2017; Ribeiro et al., 2020). The additional benefits of H_2_ adsorption, spillover, and electron transfer have been recognized for carbonaceous materials in hydrogenation reactions (Deng et al., 2009).

Meanwhile, various transition metals have also been explored as active metal sites for the hydrogenation of saccharides. Among these, noble metals such as Pt and Ru are among the most effective candidates, although their high price is a drawback (Kobayashi and Fukuoka, 2013; Negoi et al., 2014; Wang et al., 2014; Lazaridis et al., 2015).

Alternative metal phases have also been scanned to make the process more cost-competitive, ranging from non-noble metals such as Ni, Co, Fe, or Cu (Deng et al., 2009) to bimetallic compositions (Fukuoka and Dhepe, 2006; Pang et al., 2012; Frecha et al., 2019). Special attention has recently been drawn to Ni, due to the quite encouraging results that have been shown for its use. The high catalytic activity obtained from the as-produced carbon nanofibers containing Ni particles initially used for their growth should be mentioned, as they afford 56.5% hexitols after treatment with ball-milled cellulose at 190°C and 4.0 MPa H_2_ for 24 h (Van de Vyver et al., 2010). This value was further increased to 75.6% after a proper balance was attained between the acid/metal functions (Van de Vyver et al., 2012). Unfortunately, these findings cannot be transferred to the overall set of Ni catalysts, as many other authors identified rather poorer catalytic activities for the remainder of them (Kusserow et al., 2003; Fukuoka and Dhepe, 2006; Zheng et al., 2010; Wang et al., 2012b; Han and Lee, 2012; Shrotri et al., 2012; Yang et al., 2012; Zhang et al., 2018). For instance, the maximum yield to hexitols reported by Wang et al. (2012b) over various supported Ni catalysts barely accounted for 12.5%. This value was obtained from microcrystalline cellulose (95% conversion) after 2 h at 245°C and 6.0 MPa H_2_ for a 20 wt% Ni/SiO_2_ catalyst, upon the testing of many support materials (ZnO, Al_2_O_3_, ZrO_2_, SiO_2,_ TiO_2,_ MgO, AC, and kieselguhr). Similar productivity (11.6% hexitols) was attained by Zheng et al. (2010) after converting 71.9% microcrystalline cellulose on a 20 wt% Ni/AC catalyst for 30 min at 245°C. In other cases, despite relatively high yields being obtained for total polyols, low selectivity to hexitols was attained. In cases such as these, Zhang et al. (2018) Ni nanoparticles (12.5 wt%) were immobilized onto several carbons. When rigorous control of the number of meso- and/or micropores was adopted, the optimized Ni/MMC catalysts permitted the effective conversion of microcrystalline cellulose into C3-C6 polyols (45 wt%) at 240°C, 4.0 MPa H_2_, and 2.5 h. Of these, only 20% corresponded to hexitols. These discrepancies indicate that multiple variables related to the conditions of catalyst preparation and processing may concur in determining the final product spectra. A sound reason to explain these variations could be based on Ni weight requirements (Van de Vyver et al., 2012; Cao et al., 2013). Taking up these hypotheses, Sels’s group discussed the effects of metal loading (2.6–10.4 wt%) on a series of Ni/CNF catalysts, showing a similar dispersion (D) of metal and distribution (D = 1.7–2.1 wt%, 16 nm) of particle sizes. The hexitol yield increased as the Ni percentage was increased to 7.5 wt%, whereas an excessive amount of Ni promoted successive hydrogenolysis reactions. Up to 69.4% sorbitol was converted in contact with 10.4 wt% Ni/CNF under one-pot reaction conditions (24 h, 190°C, 6 MPa H_2_), with mannitol (11%) and C2-C5 molecules (37.5%) as the major degradation products (Van de Vyver et al., 2012). Taking a catalyst with low hydrogenolysis ability as a reference, Liang et al. (2012) employed a zeolite-supported Ni catalyst (Ni/ZSM-5) to illustrate the importance of Ni microstructure (morphology, crystallinity and reducibility) on hexitol selectivity. Yields of 24.7, 58.2, and 40% were noted upon a gradual increase in Ni content from 5 to 17 and 40 wt%. Under identical reaction conditions (230°C, 4.0 MPa H_2_, and 6 h), only a small portion of hexitols (2.6–8.6%) suffered from hydrogenolysis. The superior catalytic activity of Ni/ZSM-5 (17%) was ascribed to a specific petaloid-like morphology with predominantly exposed Ni (111) crystal planes and stronger support interaction. This catalyst was modified with noble metals in a later study with a nominal metal fraction of 1 wt% (M = Pd, Pt, Ru, Rh, and Ir). All bimetallic catalysts outperformed the Ni/ZSM-5 behavior (52.7% hexitols) except for Ir-Ni. The highest hexitol yield (76.9%) was obtained from the Ni-Pt/ZSM-5 catalyst after treatment of microcrystalline cellulose at 240°C and 4.0 MPa H_2_ for 4 h. The good dispersion of Pt-Ni nanoparticles (mean size of 8.4 nm *vs.* 19 nm on monometallic Ni) together with the remarkable hydrogen spillover from the alloy surface was considered able to explain this excellent hydrogenation outcome (Liang et al., 2014a). However, it remains unclear whether an increase in the metal surface area induced at smaller particle sizes could reduce the need for metal content. For example, Ribeiro and co-workers managed to produce 69.8% hexitols (61% sorbitol) after 5 h at 205°C and 5.0 MPa H_2_ on a Ru-Ni/CNT catalyst with a relatively lower metal loading (Ni:Ru = 3:0.4 wt%) (Ribeiro et al., 2017).

The nature of the support also plays an essential role in the hexitols selectivity over Ni catalysts. In this regard, the use of carbon materials was considered to be beneficial because the lack of acid-base pair sites could retard the hexitol dehydrogenation to unsaturated species, which is the step that precedes a series of concatenated transformations (retro-aldol condensation, hydrogenation, and dehydration) that end-up forming short-chain polyols (Liang et al., 2014b). However, these reactions are also sensitive to temperature and can be equally promoted under the action of the H^+^/OH^−^ ions generated from hot water dissociation (Liang et al., 2014b), an equilibrium often used to accelerate the depolymerization of microcrystalline cellulose (Akiya and Savage, 2002; Luo et al., 2007; Guo et al., 2012). In this context, with the temperature playing two antipathetic roles, the proper control of the residence time is key. Another option to explore is the pretreatment of cellulose to disrupt its crystalline structure in advance, affording its dissolution under milder conditions. For instance, the hydrothermal dissolution of microcrystalline cellulose has been reported to occur at a low extent at 190°C (35% upon 24 h) (Jollet et al., 2009), whereas up to 71.0% ball-milled cellulose was solubilized after only 16 h at the same temperature (Kobayashi, Ito, Komanoya, Hosaka, Dhepe, Kasai, Hara, Fukuoka).

In summary, relevant bibliographic reports suggest that multiple factors tend to compromise hexitol selectivity over supported Ni catalysts, in which catalyst design aspects are frequently overlaid with operational conditions and mass transport effects (primarily determined by the crystallinity index of cellulose and H_2_ gas pressure). In this work, the hydrolytic hydrogenation of cellobiose was used as a model reaction to experimentally investigate the role of the catalyst design details (metal loading and crystal size) over a series of carbon-nanofiber-supported catalysts. This reaction represents a simplified pathway whereby hydrogenation variables can be more easily discriminated, as a soluble substrate with negligible diffusion resistances is used as a feedstock (Li et al., 2013; Negahdar et al., 2014; Frecha et al., 2019). In this manner, all aspects related to hydrogenation effectiveness and catalyst preparation can be unambiguously discussed.

## 2 Materials and methods

### 2.1 Catalyst preparation

#### 2.1.1 Support synthesis

Fishbone-type carbon nanofibers were grown in a rotary bed reactor using the catalytic decomposition of biogas (CDB) over a Ni-Co/Al_2_O_3_ (Ni:Co:Al = 33.5:33.5:33 mol%) catalyst. In brief, 5.0 g of fresh catalyst was placed in the reactor and reduced *in-situ* by a H_2_ stream (80 ml STP/min) for 1 h at 600°C. Next, the CDB reaction was carried out at 650°C for 3 h, feeding a synthetic equivolumetric mixture of CH_4_:CO_2_ (1:1 vol./vol.) at a gas hourly space velocity of 30 L·(g_cat_·h)^−1^. Additional details regarding the catalyst preparation, reactor configuration, and experimental set-up can be found in the previous work of this group (Pinilla et al., 2017). Raw nanofibers are commonly functionalized through a two-step procedure, first in HCl (37 wt%, Fluka, 50 ml/g_CNF_) under ultrasonic vibration (60°C, 4 h) and then refluxed in HNO_3_ (65 wt%, Panreac, 25 ml/g_CNF_) for 1 h at 130°C. In both cases, acid-treated CNF was recovered from the liquid solution by vacuum filtration, rinsed with deionized water to neutral pH and dried at 70°C overnight. For simplicity’s sake, oxidized carbon nanofibers are hereafter called CNF.

#### 2.1.2 Metal deposition onto CNF

Three impregnation techniques were used to prepare CNF-supported Ni catalysts with various particle sizes. The nominal metal content was modulated for each sample to compensate for the available Ni surface:

• Homogeneous Deposition-Precipitation (DP): Ni(OH)_2_ nanoparticles were precipitated from an aqueous solution (150 ml, Milli-Q water, 0.055 μS/cm) of nickel nitrate (Ni(NO_3_)_2_·6H_2_O, Alfa Aesar, 98%) by means of pH changes, following the method described elsewhere (Romero et al., 2017). In practice, the suspension was basified to pH = 8.2 with ammonia (NH_4_OH, Panreac, 30 vol%) and was maintained in contact with the support (CNF, 1.35 g) under mild stirring (200 rpm) for 30 min at 40°C (Nieto-Márquez et al., 2010). The precipitate was then gathered using vacuum filtration, washed with deionized water to neutral pH, and dried at 60°C overnight. The amount of dissolved nickel nitrate varied between 0.83 and 1.27 g according to the desired metal loading (10–14 wt%), assuming a theoretical Ni deposition percentage of 90%.
• Dry impregnation (DI): Following this procedure, the salt precursor was dissolved in just the amount of liquid needed to fill the pore volume of the catalyst support (2.7 ml/g CNF, experimentally determined). In this case, an aqueous solution (9.45 ml) of nickel nitrate (1.9661 g, Ni(NO_3_)_2_·6H_2_O, 98%, Alfa Aesar) was drop-wised to the support (3.5 g CNF), which was then dispersed ultrasonically for 10 min and dried at 60°C overnight.
• The grafting of pre-reduced Ni nanoparticles onto CNF (Ni NP): Uniform-sized Ni nanoparticles (monodispersed) were synthesized using a colloidal-deposition approach (Toebes et al., 2003). Broadly, the synthesis consisted of an *ex-situ* reduction of metal colloids in the organic medium, followed by their self-assembly onto the carbon material. Then, the metal surface was cleaned from adventitious organic ligands by air oxidation and was reduced to regain the metallic state. A schematic illustration of the experimental set-up is depicted in Supplementary Figure S1. In a typical synthesis, Nickel II acetate (1.24 g, Ni(ac)_2_·4H_2_O, Sigma Aldrich, 99.8%) was dissolved in a mixture of oleylamine (OAm, 75 ml, 70%, Aldrich) and oleic acid (OA, 1.6 ml, 99.5%, Aldrich) inside a three-necked flask (250 ml) connected to a N_2_ line. The solution was first degassed and dehydrated at 110°C for 1 h under stirring (950 rpm), affording a green Ni-oleate complex. At this point, the solution was cooled down to 90°C and then reduced by a fast injection of borane trimethylamine (Et_3_N·BH_3_, 2.5 ml, Fluka, 95%) and further aged for 1 h. Once at room temperature, the oleylamine excess was dissolved in a double volume of ethanol (150 ml, Panreac, 96%). Black Ni NPs were finally collected by centrifugation (8,500 rpm, 8 min), re-dispersed in hexane (300 ml) and grafted onto CNF (4.5 g). To ensure complete adherence to the support, the colloidal mixture was stirred for 3 days (450 rpm) at room temperature. Lastly, the catalyst was dried by rotary evaporation under a vacuum, and the metal surface was activated by thermal annealing at 185°C for 5 h in the air (40 ml/min) (Serp et al., 2003). This cleaning stage, although necessary to remove organic ligands around the metal surface, modified the metal state of the Ni, and a second reduction stage with H_2_ was required (Supplementary Figure S2).

Fresh catalysts were finally reduced in a tubular furnace (750 × 15 mm). For this purpose, the samples were thermally treated for 1 h at 450°C (heating rate of 10°C/min) under an inert atmosphere (75 ml/min N_2_, 99.9992%, air liquid) and subsequently reduced with H_2_ (100 ml/min, 99.9992%, air liquid) at the same temperature for another 2 h. After cooling to room temperature, the metal surface was passivated overnight by an oxygen-limited stream (O_2_/N_2_, 1:99 vol./vol.; 40 ml STP/min, air liquid). The reduction temperature was defined on the basis of the TPR pattern for Ni/CNF_DI_, depicted in Supplementary Figure S3. A similar reduction range was reported for the catalyst prepared by deposition-precipitation in the literature (Nieto-Márquez et al., 2010). CNF-supported Ni NP was simply treated in a H_2_ flow at 300°C for 1 h, according to their TPR profile (Supplementary Figure S3) and then passivated.

### 2.2 Characterization techniques

The supported metal catalysts were characterized using different instrumental techniques. These include temperature-programmed reduction (TPR) and thermogravimetric analyses for reducibility studies, X-ray photoelectron spectroscopy (XPS) for metal valence state, and actual metal loading by inductively coupled plasma-optical emission spectroscopy (ICP-OES). The morphology of nanoparticles and their crystalline features were studied by transmission electron microscopy (TEM) and X-ray diffraction (XRD) analyses, respectively.

TPR-H_2_ experiments were performed on an AutoChem II 2920 station by Micromeritics. For these tests, fresh catalyst (200–250 mg), previously stabilized at 110°C by an inert gas, was subjected to a gradual heating (10°C/min) from 45°C to 500°C under a H_2_ stream (H_2_/Ar, 10:90 vol./vol., 50 cm^3^/min) while a thermal conductivity detector (TCD) recorded variations in the outlet gas stream composition. The peaks in hydrogen consumption were related to the regions of metal reduction. In some instances, the TPR-H_2_ profile was acquired on a NETZSCH TG 209 F1 Libra thermal analyzer, coupled with mass spectroscopy. The analysis was performed with an initial mass of around 8 mg, increasing the temperature from 30°C to 600°C at a rate of 10°C/min in a reductive controlled environment (H_2_/N_2,_ 10% vol./vol., at a constant flow of 50 ml STP/min). The outgoing gas was continuously monitored by a mass spectrometer (quadrupole OmniStar TM, Pfeiffer).

The XPS measurements were carried out in an ESCAPlus OMICROM System, equipped with a hemispherical electron energy analyzer, an Al/Mg dual anode, and a detector with seven channeltrons. The spectrometer was operated at 225 W (15 mA and 15 kV), using a non-monochromatic Mg Alα radiation (Kα = 1,253.6 eV) as the excitation source. Survey scans were acquired at a constant pass energy of 50 eV in the range of 1,000 and 0 and 20 eV for higher-resolution spectra of individual regions. The base pressure in the chamber was maintained below 5 × 10^−9^ Torr. For the spectra processing, Casa^®^XPS software was used, fitting the signals with a Voigt Gaussian-Lorentzian (GL60) line shape and a Shirley-type background. The binding energy values were calibrated with reference to the graphitic C1s position (284.6 eV) within ±0.2 eV accuracy. The sensitivity factors, corrected for escape depth, were supplied by the instrument manufacturer.

A SPECTROBLUE (Ametek) spectrometer was employed for the ICP-OES determinations. Each sample was digested by sodium peroxide (Na_2_O_2_) according to the fusion method. This technique was similarly applied to check possible metal leaching into the reaction solution.

X-ray diffractograms were acquired in the θ-θ configuration (scanned range of 5–80°, step size = 0.05°, counting time/step = 4 s) using a Bruker System (Model D8 Advance, Series 2), with a Cu K_α_ anode (λ = 1.54056 Ȧ, 40.0 kV, 20.0 mA) and a secondary graphite monochromator as the radiation source. The ICCD database and DIFRAC PLUS EVA 8.0 (Bruker) software were used for phase assignments and XRD data processing, respectively.

TEM micrographs were taken with a field emission-scanning electron microscope (Tecnai F30, FEI company) at a filament voltage of 300 kV. The instrument is equipped with SuperTwin^®^ lenses, allowing a maximum point resolution of 1.5 Å. Before the analysis, the samples were ultrasonically dispersed in ethanol. A droplet of this suspension was deposited onto a holey copper grid coated with a lacey carbon film.

Particle size distribution analysis was performed using ImageJ software from TEM measurements, and at least 150 metal nanoparticles were counted.

The specific surface area of the nickel (S_Ni_) was estimated from the size distribution of the metal particles measured profiles taking into consideration the geometry of the particles, the Ni density (ρ_Ni_ = 8.9 g/cm^3^), and the value for the of surface area-weighted diameter ( ${\bar{d}}_{s}$ ), following the mathematical expressions in Eqs. 1 and 2 (Nieto-Márquez et al., 2010; Romero et al., 2017):

$$S_{Ni}=\frac{6}{\rho_{Ni}\cdot {\bar{d}}_{s}}$$ (1)

$${\bar{d}}_{s}=\frac{\sum_{i}n_{i}\cdot d_{i}^{3}}{\sum_{i}n_{i}\cdot d_{i}^{2}}$$ (2)

In Eq. 2, n_i_ is the number of particles with a given diameter (d_i_).

The nickel surface area per unit mass of the catalyst (S) was then determined from the specific surface area of Ni and the bulk metal content measured by ICP-OES (Eq. 3):

$$\text{S}={\text{S}}_{\text{Ni}\cdot }\cdot \left(\frac{\text{wt}.\text{ \% Ni}}{{\text{g}}_{\text{catalyst}}}\right)$$ (3)

### 2.3 Reactor set-up

The hydrolytic hydrogenation reaction was studied in batch mode in a high-pressure autoclave reactor (Parker Autoclave Engineers, 100 ml) that was equipped with a PID controller and a magnetic stirrer. In a typical set-up, 300 mg cellobiose (purity >98%, Sigma Aldrich^®^, moisture content about 3 wt%), 150 mg catalyst and deionized water (30 ml) were loaded into the reactor. The apparatus was then sealed, purged, and vented with N_2_ and H_2_, in that order. Next, the autoclave was pressurized to 4.0 MPa of H_2_ (measured at room temperature) and heated to 190°C under mild stirring (300 rpm). Zero time was considered to occur when the set-pointed temperature was reached, once the stirring rate raised to 1,000 rpm. At the end of the test (180 min later), the reactor was quenched, depressurized, and opened. The spent catalyst was recovered by vacuum filtration (cellulose, 0.22 µm, Whatman^®^), washed with water to desorb possible retained products and dried overnight (70°C). To ensure experimental reproducibility, reaction tests were performed in duplicate. The results correspond to average values expressed as means ± standard deviations.

### 2.4 Product analysis

The analysis of the water-soluble products was performed using high-performance liquid chromatography (HPLC). The system used, the LC-2000 Plus Series by Jasco, is fitted with a semi-micro HPLC pump PU-2085, a refractive index detector (Jasco RID-2031) and a strong cation-exchange resin column (Reprogel Pb, 9 μm, 8 × 300 mm, ReproGel^®^, Maisch) preceded by a guard column. The column was operated at 80°C using ultrapure H_2_O (0.055 μS/cm) at a flow rate of 0.5 ml/min as the eluent. The sample separation (volume of 50 μl) was complete within a run time of 56 min, holding the cell temperature at 30°C. Analogous conditions were kept with the analytical standards used in the quantification by the external standard method. Routinely, the solution was re-filtered before the injection through a 0.45 μm PTFE syringe filter.

The mass balance included the possible formation of gaseous products (CO_2_, CO, CH_4_) from outlet gas samples analysis. The analysis of the gas phase was carried out on a Micro GC (Varian CP4900) equipped with two packed columns (Molecular Sieve and Porapack) and a TCD detector. The calibration of compounds was performed daily by interpolation from area peaks using a reference gas containing 15% CO_2_, 20% CH_4_, 30% CO, and 35% H_2_ (expressed in vol%).

The conversion of cellobiose was estimated as the difference between the inlet concentration and the one determined by HPLC after reaction:

$$\;X_{CELLOBIOSE}\;\left(\%\right)=\left(1-\frac{mass\;of\;unreacted\;cellobiose}{mass\;of\;cellobiose\;fed}\right)\cdot 100$$ (4)

The product’s yield (Y) was expressed in wt% and calculated as the ratio between their mass and the initial mass of cellobiose:

$$Y\;\left(\%\right)=\left(\frac{\;product\;mass}{mass\;of\;substrate}\right)\cdot 100$$ (5)

## 3 Results and discussion

### 3.1 Support characterization

**FIGURE 1:**
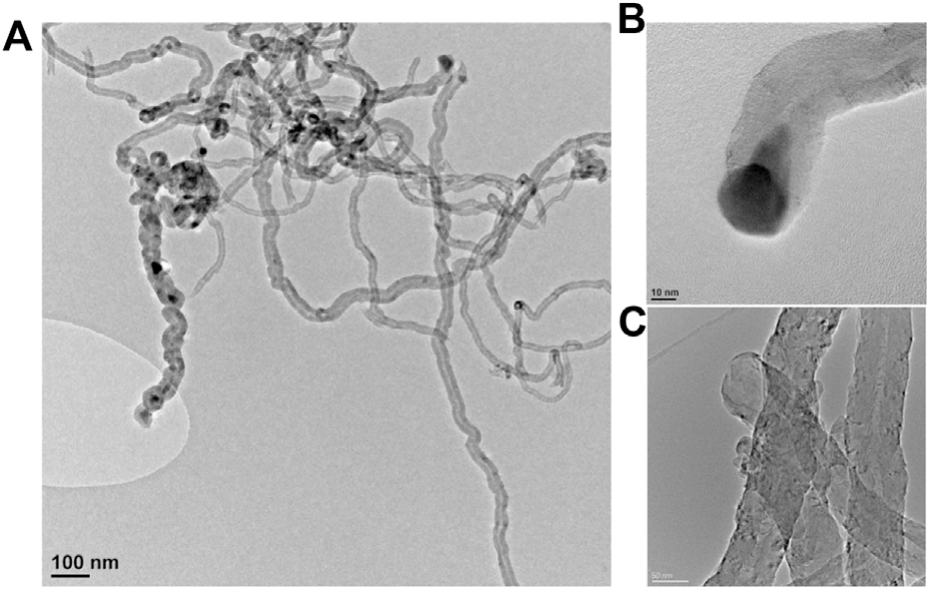
TEM images of CNF before **(A,B)** and after purification **(C)**.

**FIGURE 2:**
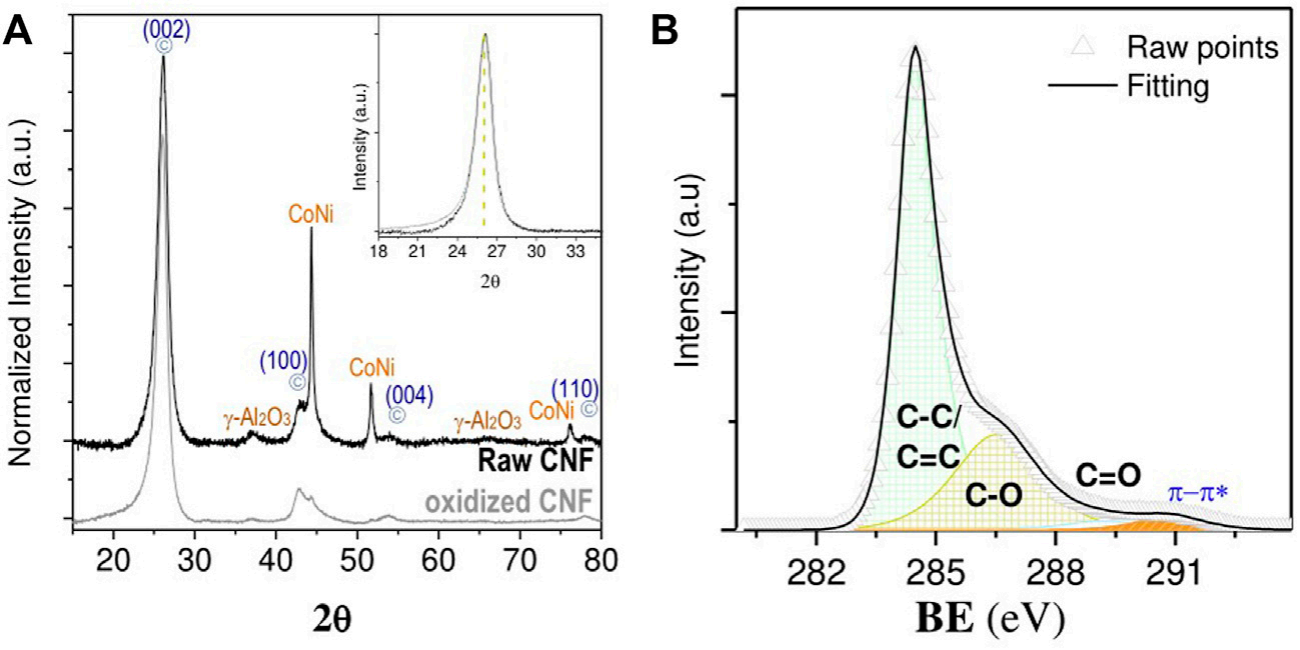
XRD pattern of raw and oxidized CNF **(A)** and C1s XPS spectra for oxidized CNF **(B)**.

The CNF used as a catalytic support exhibits an entangled morphology, forming porous bundles in the micrometer range (Figure 1). Before purification, the raw CNF held the metal catalyst used for the growth at the tip, arranged as elongated pear-shaped clusters of Ni-Co/Al_2_O_3_, whose sizes coincided with the filament diameter (Figures 2B). On the whole, the catalyst content was 9.3 wt% (3.1 wt% Ni, 3.2% Co, and 1.6% Al in the form of Al_2_O_3_, measured by ICP-OES).

The XRD patterns of the raw CNF exhibited characteristic reflections of graphite-like structures, along with metal signals of Ni-Co catalysts (Figures 2A). The metal signals vanished after the subsequent treatment in acid, whereas the carbon diffraction lines remained unchanged, as shown in the inset of Figures 2A. These results confirm that the two-step purification successfully eliminated the vast amounts of metals with no obvious impact on the structural integrity of the CNF. Only minute amounts of metal residue (0.54 wt% Ni, 0.41% Co, and 0.67% Al) persisted still embedded into the acid-treated CNF, as measured by ICP-OES.

The activation of CNF by an oxidative stage simultaneously creates various oxygen-containing surface groups onto the CNF backbone, which were earlier recognized as useful anchoring points for metal precursors during the impregnation process (Serp et al., 2003; Toebes et al., 2003). Surface chemistry analysis by XPS revealed that most oxygen atoms involve C-O-C species (286.5 eV), such as hydroxyl/epoxides groups, among other carbonyl/carboxylic groups (O-C=O, 288.5 eV), which appeared in minor proportions (Figures 2B).

### 3.2 Characterization of catalysts

**FIGURE 3:**
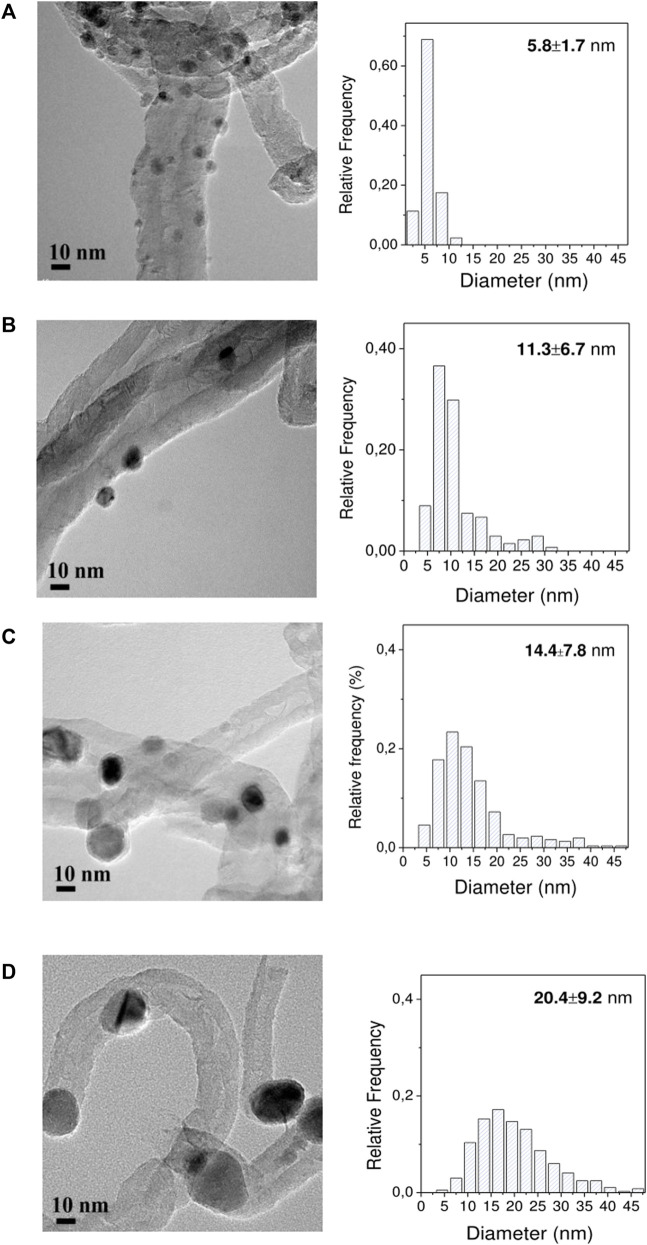
HRTEM images and derived particle size histograms: **(A)** Ni NP/CNF, **(B)** Ni/CNF_DI_, **(C)** 7.3% Ni/CNF_DP_, and **(D)** 14% Ni/CNF_DP_.

The three impregnation methods (precipitation in basic media, incipient wetness impregnation, and self-assembly of *ex-situ* pre-synthesized nanoparticles) rendered Ni catalysts that had different crystal sizes as measured by TEM (Figure 3) and metal loadings by ICP (Table 1). Nearly monodispersed nanoparticles of small dimensions (5.8 ± 1.7 nm) were prepared from the surfactant-mediated synthesis (Ni NP), achieving a final Ni loading of 5 wt%. On the other hand, broad size distribution and large mean diameter were shown for the Ni/CNF_DP_ (14.4 ± 7.8 nm) with a Ni loading of 7.3 wt%. Medium-size particles were obtained from the Ni/CNF_DI_ catalyst (11.3 ± 6.7 nm, 10.7 wt% Ni).

**TABLE 1:** Physicochemical properties of various CNF-supported Ni catalysts: Ni loading by ICP, particle diameter (d_p_) from XRD and TEM characterization, surface-area weighted diameter ( ${\bar{d}}_{s}$ ), Ni surface area per metal gram (m^2^/g_Ni_), and catalyst mass unit (m^2^/g _cat_) and oxidation state ratio according to XPS.

Sample	Ni loading^a^ (wt%)	d_p_ (nm)	d_p_ (nm)	${\bar{d}}_{s}$ (nm)	S_Ni_ (m^2^/g_Ni_)	S (m^2^_Ni_/g_cat_)	Ni^0^/(Ni^II^ + Ni^0^)
Ni NP/CNF	5.0	---	5.8 ± 1.7	6.8	99.1	4.42	5.4%
Ni/CNF_DI_	10.7	12.5	11.3 ± 6.7	17.6	38.3	3.89	9.2%
Ni/CNF_DP_	7.3	20.3	14.4 ± 7.8	22.0	30.7	2.13	12.6%
Ni/CNF_DP_	14.0	24.1	20.4 ± 9.2	26.2	25.7	3.46	22.4%

a Including 0.54 wt% Ni from CNF support. This value was excluded from the characterization results.

The analysis of particles’ sizes gives an indication of their geometrical areas, which is an indirect measurement of the total surface area available for catalysis. Approximate but applicable calculations can be performed using TEM measurements with surface-area weighted diameter ( ${\bar{d}}_{s}$ ) and metal density (*ρ*_*Ni*_ = 8.9 g/cm^3^), assuming a hemispherical form. As shown in Table 1, the surface exposure of Ni per unit mass (m^2^/g_Ni_) decreased with increasing the mean size of particles as follows: 99.1 ( ${\bar{d}}_{s}$ = 6.8 nm) < 38.3 ( ${\bar{d}}_{s}$ = 17.6 nm) < 30.7 ( ${\bar{d}}_{s}$ = 22.0 nm) for Ni NP/CNF, Ni/CNF_DI_, and Ni/CNF_DP_, respectively. In catalysis, the reduction in the surface to volume ratio derived from the use of larger particles must be compensated with higher bulk metal concentrations to ensure a minimum number of catalytic sites. These values served as a reference to further adjust the metal Ni loading in the DP catalyst (which only showed a Ni specific surface area per mass unit of 2.13 m^2^/g_cat_ at 7.3% Ni loading) until a Ni surface area comparable to those of Ni NP/CNF and Ni/CNF_DI_ (4.42 and 3.89 m^2^/g, respectively) was attained. Thus, a second 14 wt% Ni catalyst was prepared by the deposition-precipitation method. The increment in the metal content from 7.3 to 14.0 wt% led to particles that were a bit larger (14.4 *vs.* 20.4 nm, determined by TEM) and a Ni specific surface area at *ca*. 60% (3.46 m^2^/g). As a result, three catalysts bearing 3.92 (±0.47) m^2^ Ni per gram of sample were prepared when bulk metal fractions of 5, 10.7 and 14 wt% were used to synthesize the NPs Ni/CNF, Ni/CNF_DI_, and Ni/CNF_DP_ catalysts, respectively.

**FIGURE 4:**
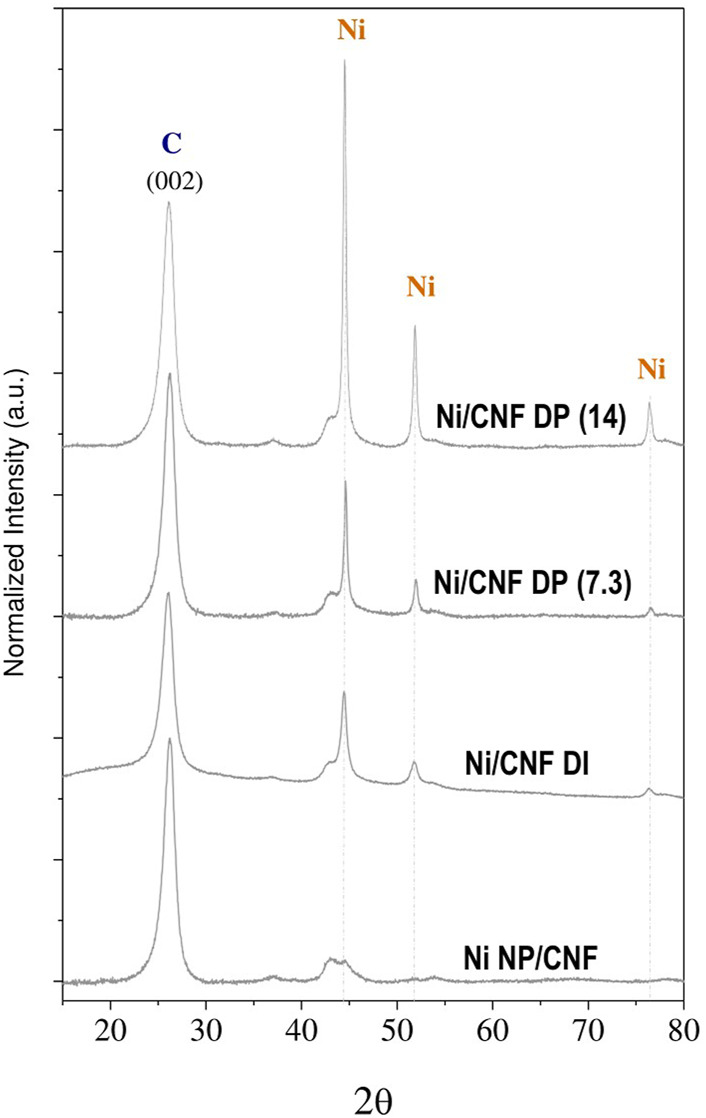
X-ray diffraction pattern for the set of Ni/CNF catalysts.

The crystalline nature of the metal phase was examined by XRD (Figure 4). Clear diffraction lines, centered at 2θ = 44.5°, 51.8°, and 76.3°, were noticed for Ni/CNF_DI_ and Ni/CNF_DP_ samples, which were indexed to the (111), (200), and (220) crystallographic facets of Face-Centered Cubic (FCC) nickel, respectively. The shape and size of the Ni (111) peak defined a mean crystallite size at around 12.5, 20.3, and 24.1 nm (Table 1), based on the Debye-Scherer’s formula. These values lie within the size range quoted from the TEM analysis. No Ni reflections were shown by Ni NP/CNF catalyst, consistent with smaller particles and lower metal contents. Nor characteristic diffraction lines of the oxidized phase were observed, revealing the absence of metal oxide crystallites larger than 5 nm in any case. The broad signal at 2θ = 26.09°, signature of the (002) graphitic plane, as well as other characteristic minor peaks of the support at 2θ = 42.5° (100), 44.3° (101), 53.9° (004), and 78° (110) could be distinguished over the entire set of samples.

**FIGURE 5:**
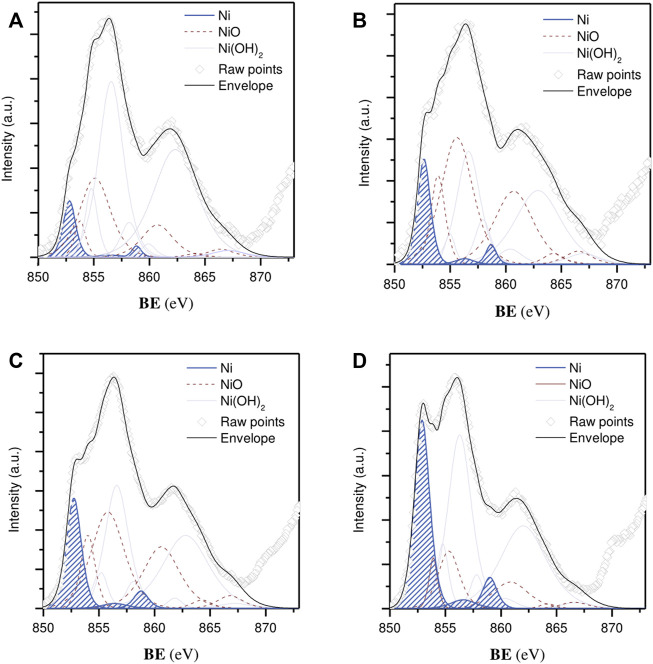
Ni 2p 3/2 core level spectra: **(A)** Ni NP/CNF, **(B)** Ni/CNF_DI_, **(C)** 7.3% Ni/CNF_DP_, and **(D)** 14% Ni/CNF_DP_. Metallic Ni state contributions are marked in blue.

To gain further information about the oxidation state of Ni species at the near surface, X-ray spectroscopy (XPS) measurements were performed (Figure 5). The Ni 2p curve fitting was resolved with multiple splittings, shakings, and plasmon loss structures, whose empirical fitting parameters (i.e., peak positions and spacing, FWHM and area percentages) were taken from the literature (Biesinger et al., 2009). A spin-orbit splitting distance of 17.3 eV was sufficiently large to only consider the more intense component (2p 3/2) within the line-shaped envelope. The width of this region was broad, showing contributions from both metallic and oxidized species in all cases. Three main peaks could be used to isolate reduced forms from such a complex spectral profile, including a major signal (79.6%) at a BE of 852.6 eV and twinned satellites, positioned at 856.3 and 858.7 eV, with a relative area contribution of 5.6% and 14.8%, respectively. Accordingly, the proportion of surface Ni atoms corresponding to the active phase (Ni^0^/(Ni^II^ + Ni^0^)) rose from 5.4% to 9.2% and from 12.6% to 22.4% for the Ni NP/CNF, Ni/CNF_DI_, and Ni/CNF_DP_ catalysts, respectively (Table 1). The remainder of the Ni fraction belonged to an almost overlapped distribution of various oxidized states (i.e., NiO, Ni(OH)_2_).

### 3.3 Catalytic activity

**FIGURE 6:**
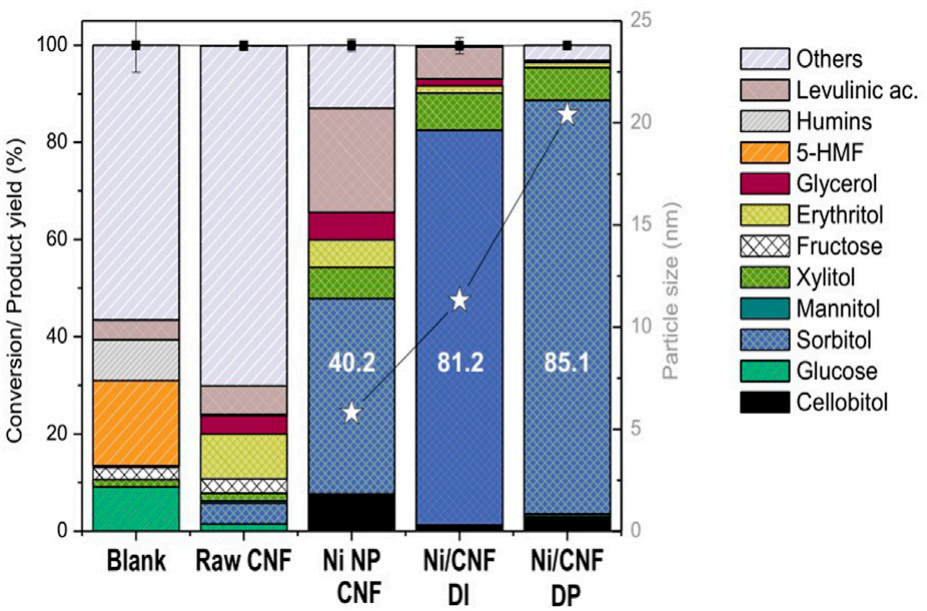
Catalytic results for hydrolytic hydrogenation of cellobiose. Error bars indicate ±standard deviations (*n* = 2).

The hydrolytic hydrogenation of cellobiose was assessed at 190°C under 4.0 MPa H_2_ for 3 h. The main catalytic results obtained at the end of the tests are graphically displayed in Figure 6, while the specific product distribution is listed in Table 2.

**TABLE 2:** Cellobiose conversion and product distribution using various Ni-based catalysts.

Entry	Catalyst	X (%)	Y_PRODUCT_ (mass%)							
			Sugars^a^	Levulinic acid	Sugar alcohols^b^					Others^c^
					C_12_	C_6_	C_5_	C_4_	C_3_	
1	Blank	100	11.5	4.0	n.d.	n.d.	1.5	n.d.	0.3	82.7
2	Raw CNF	99.9	4.5	5.9	n.d.	4.8	1.6	9.3	3.8	70.0
3	Ni NP/CNF	100	0.4	21.5	7.3	40.2	6.5	5.5	5.7	12.9
4	Ni/CNF_DI_	99.9	0.3	6.6	1.0	81.2	7.7	1.5	1.5	0.1
5	Ni/CNF_DP_ (14 wt%)	99.9	0.5	n.d.	1.8	85.2	6.7	1.0	0.2	4.5
6	Ni/CNF_DP_ (7.3 wt%)	99.9	0.3	17.5	2.9	49.8	6.7	5.1	4.3	13.3

a Sum of glucose and fructose.

b C_12_ = cellobitol, C_6_ = sum of sorbitol and mannitol, C_5_ = xylitol, C_4_ = erythritol, C_3_ = glycerol.

c Including 5-HMF, humins, and undefined products.

n .d. = not detected.

**SCHEME 2:**
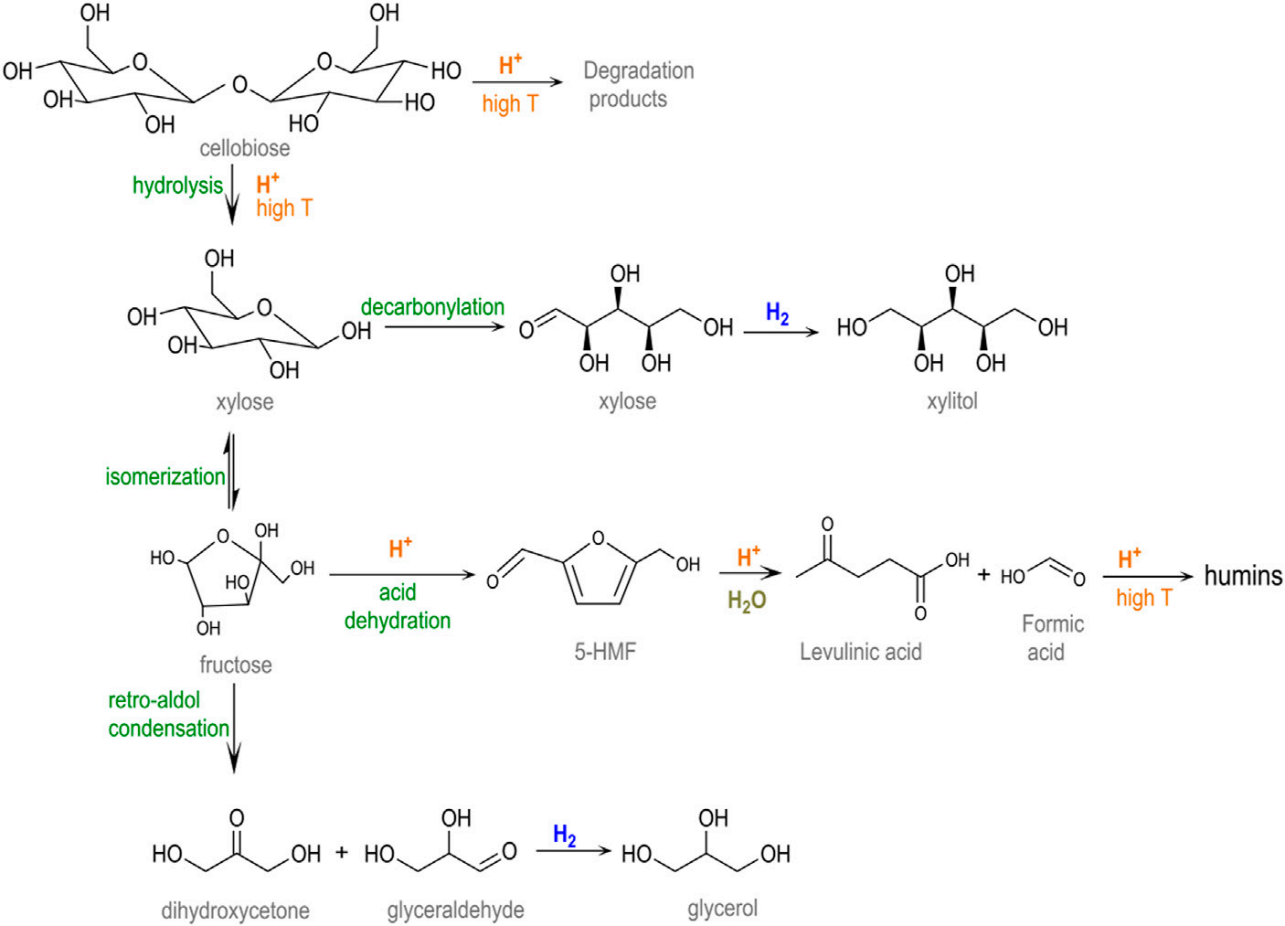
Reaction network involved in the cellobiose hydrolysis (adapted from Lazaridis et al. (2017) and Hausoul et al. (2015)).

Experimental results reveal that a nearly complete conversion of cellobiose (>99.9%) was attained for a non-catalyzed hydrothermal treatment (blank test). In these conditions, the acidity of the system generated from the auto-hydrolysis of water at a high temperature was enough to thoroughly hydrolyze the β-1,4-glycosidic bonds. Nonetheless, only a small percentage of glucose was recovered from the liquid solution (9.1%), most of it being thermally decomposed (90.9%). Scheme 2 depicts the major pathways for glucose degradation under hydrothermal conditions (Hausoul et al., 2015; Lazaridis et al., 2017). In particular, a small fraction of sugars (2.5%) were isomerized to fructose, which further underwent dehydration reactions to 5-hydroxymethyl furfural (5-HMF, 17.6%). This compound was either not stable in aqueous media, being readily hydrolyzed into levulinic (4.0%) and formic acids and finally condensed to furan-based insoluble substances (humins, 8.4%). Almost no hydrogenation products were detected in the blank test, excluding trace amounts of xylitol (1.5%) and glycerol (0.3%) that presumably came from sugars retro-aldol reactions and subsequent hydrogenations. The rest of the degradation products (56.6%) remained unidentified. The extensive formation of by-products under non-catalytic conditions is commonly reported for this reaction and is ascribed to the presence of a chemically unstable aldehyde group (Kobayashi et al., 2014).

The catalytic activity of the Ni-Co/Al_2_O_3_ particles attached to the CNF before the purification stage was also checked (CNF, Entry 2). Despite their distinguished morphological features (i.e., particles are reshaped during the catalytic decomposition of biogas reaction and utterly dispersed) (Duan et al., 2015), no important hydrogenation activity was attained (4.8% hexitols; 4.25% sorbitol). Small molecular weight polyols were also detected in small quantities, involving xylitol (1.6%), erythritol (9.2%), and glycerol (3.8%) as the most representatives. This result differs from previous studies found in the literature that report 56.5% hexitols from ball-milled cellulose on a similar 3 wt% Ni/CNF catalyst, not less because most of the metal moieties remained inaccessible for catalysis by being occluded between graphene layers (Van de Vyver et al., 2010). This last point is visually verifiable by electron microscopy of individual particles (Supplementary Figure S4). Another argument to explain such differences in the catalytic behavior could be based on metal phase composition changes, including an additional fraction of Co in equimolar proportions (Ni-Co alloy, 50:50, 6.3 wt%). However, significantly high sorbitol production (yield of 86%) was equally reported for a HZSM-5 zeolite supported Ni-Co alloy (Ni:Co = 10:5 wt%) at the complete conversion of cellobiose (180°C, 5.0 MPa H_2_, 5 h) (Zada et al., 2018).

Very differently, the additional incorporation of Ni onto the CNF (Ni NP/CNF, Ni/CNF_DI_ and Ni/CNF_DP_) resulted in an excellent stabilization of sugars, affording overall mass recoveries higher than 87.1% and a product distribution mainly composed of sorbitol. Supplementary Scheme S1 summarizes the main reaction routes involved in the catalytic conversion of cellulose to sorbitol. In addition to sorbitol, other by-products were detected in the reaction media, including smaller molecular polyols, such as xylitol, erythritol and glycerol *inter alia*. The sum of C5-C3 polyols ranged between 7.9% and 17.7%, increasing for catalysts with lower hydrogenation activity. Likewise, glucose degradation in levulinic acid became notorious as the hydrogenation rate decayed, accounting for up to 21.5 wt% for Ni NP/CNF and to a lesser extent (6.6%) on Ni/CNF_DI_.

Despite fairly close values on their Ni surface area (∼3.92 m^2^/g_cat,_ ɛ = 12.2%), significant differences in the catalytic results were noticed from the three tested catalysts, pointing to an apparent correlation between the sorbitol production and the crystal size (Figure 6). In particular, the sorbitol yield markedly enhanced from 40.2 to 81.2 wt% when the average Ni size (defined by TEM) increased from 6 to 11 nm, followed by a slight increase to 85.1 wt% with further enlarging of the crystal size to 20 nm. Herein, the catalytic activity seemed not to be solely controlled by the availability of surface atoms but instead suggests a different reactivity of the surface atoms at various crystal sizes. The rationale behind this could be tentatively linked to the oxidation state of the metal phase due to the fact that small Ni particles are more susceptible to surface oxidation upon air contact (Kobayashi et al., 2014). This interpretation is consistent with the metal speciation proposed from XPS results, where the tiniest particles (the less active) retained a lower proportion of metallic Ni atoms at their surface layer, whereas samples with larger particle sizes (more active) were mainly reduced. The above observations suggest the use of larger Ni particles (>11 nm) to preserve the metallic character, i.e., the active phase for the hydrogenation reaction. Similar conclusions were drawn by Fukuoka et al. in the range of 2.7–16 nm (by XRD) on higher Ni loadings (10–70 wt%) (Kobayashi et al., 2014).

**FIGURE 7:**
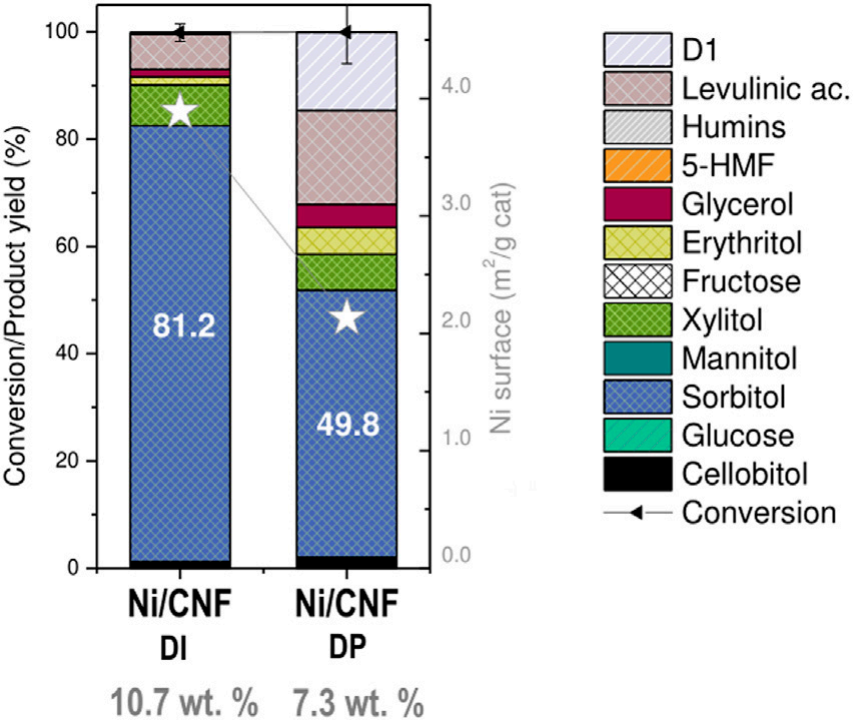
Effect of Ni loading on hydrogenation performance over Ni/CNF. Error bars indicate ±standard deviations.

It must be remarked, however, that the positive catalytic effect derived from the use of larger particles is by no means counterpoised by the use of lower Ni contents. To illustrate this point, the catalytic performance of a catalyst with a mean Ni crystal size of 14.4 nm, loaded at 7.3 wt%, was compared against the former Ni/CNF_DI_ (11.3 nm, 10.7% Ni). In this case, an increment in the particle size from 11.3 to 14.4 nm was on par with a proportional rise in the atomic surface fraction of metallic Ni from 9.2% to 12.6% (Table 2). However, a reduction in the Ni bulk loading from 10.7% to 7.3% dropped the metallic interfacial area from 3.89 to 2.13 m^2^/g_cat_ and its ulterior formation to sorbitol (81.2% down to 49.8%; Figure 7). In parallel, the decrease in the hydrogenation rate prompted the formation of other by-products from secondary off-path routes. The two most well-known examples included shorter polyols (6.7% xylitol, 5.1% erythritol, and 4.3% glycols) via retro-aldol reactions and levulinic acid (17.5%) from sugars degradation pathways.

A final issue in metal-supported catalysts relates to a possible Ni leaching into the aqueous phase (checked by ICP-OES). In all cases, a small percentage of the original Ni was dissolved into the reaction solution (5.26%, 2.33%, and 2.11% for Ni NP/CNF, Ni/CNF_DI_, and Ni/CNF_DP_ (14wt% Ni), respectively). Hence, a subsidiary advantage of using larger Ni particle sizes may relate to their lower tendency to metal leaching, although the role of the preparation method for the catalyst cannot be discarded.

## 4 Conclusion

Hydrolytic hydrogenation mediated by CNF-supported Ni catalysts arises as an attractive chemical route to control the sugars reactivity during the valorization of cellulose. However, the rational control of the distributions of reaction products requires a detailed understanding of the operating conditions, catalyst composition, and mass transport limitations. The influences of the processing conditions and catalysts composition can be isolated from that solid-solid diffusional (mass transfer) using cellobiose (a glucose dimer) instead of cellulose (a glucose polymer). In this work, correlations between Ni metal loading (5–14 wt%) and particle size (5–20.4 nm) regarding the catalytic performance were established using the *one-pot* conversion of cellobiose as hydrogenation test (190°C, 3 h) and carbon nanofibers as the metal carrier. The experimental results showed that the design parameters of higher metal loadings and larger Ni crystals were key to ensuring the availability and reactivity of Ni atoms on the catalyst surface. An optimum sorbitol yield (81.2%) was found for catalysts prepared by the dry impregnation method (10.7 wt%, mean diameter of 11.3 nm). Taken together, the results of this study could be further extended to the conversion of larger carbohydrates, putting the rational basis on the use for the synthesis of sugar alcohols from renewable resources.

## Data Availability

The raw data supporting the conclusion of this article will be made available by the authors, without undue reservation.
