# Supplementary material for: Design of highly active Ni catalysts supported on carbon nanofibers for the hydrolytic hydrogenation of cellobiose
Source: Front Chem. 2022 Aug 24;10:976281. doi: 10.3389/fchem.2022.976281 (PMC9449348; doi:10.3389/fchem.2022.976281)
Supplement: Supplementary file 1 [file DataSheet1.docx]

Supplementary Material

**Supplementary Figure 1.** Typical steps for the synthesis of Ni catalysts following colloidal-deposition approaches.

**Supplementary Figure 2.** TG-H_2_ profile for NPs Ni/CNF after removing the organic surfactants by air-oxidation (185 ºC, 5 h).

**
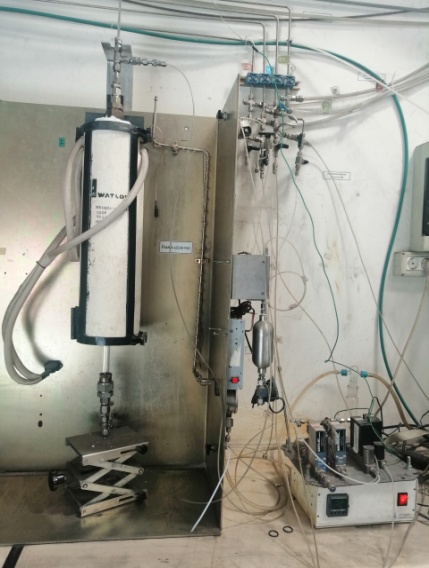
**

**B**

**A**

**C**

**Supplementary Figure 3.** Details for catalyst reduction A) Reduction reactor, B) Typical temperature program used for metal catalyst activation and C) TPR-H_2_ profile for Ni/CNF prepared by dry impregnation (10 wt. %).









**Supplementary Figure 4.** Representative TEM micrographs of raw CNF with occluded metal nanoparticles.

**Supplementary Scheme 1.** Reaction network involved in the catalytic conversion of cellobiose to sorbitol.
